# Editorial: TSH Receptor and Autoimmunity

**DOI:** 10.3389/fendo.2019.00019

**Published:** 2019-01-30

**Authors:** Terry F. Davies, Rauf Latif

**Affiliations:** Thyroid Research Unit, The Mount Sinai Medical Center and the James J. Peters VA Medical Center, Icahn School of Medicine at Mount Sinai, New York, NY, United States

**Keywords:** TSHR (thyroid-stimulating hormone receptor), GPCR (G-protein-coupled receptors), autoantibodies, TSH (thyroid stimulating hormone), small molecule

## Introduction

Over time it has become clear that the fascination with the TSH receptor (TSHR) is not only its complexity and its relationship to human disease but the fact that it keeps teaching us fundamental biology at all levels; cellular, molecular, and genetic. There are good examples of each of these facets in this cutting edge collection of papers. This contribution provides a brief and broad overview highlighting those areas of active progress by briefly eluding to some of the contributions in this collection.

**Figure 1:**
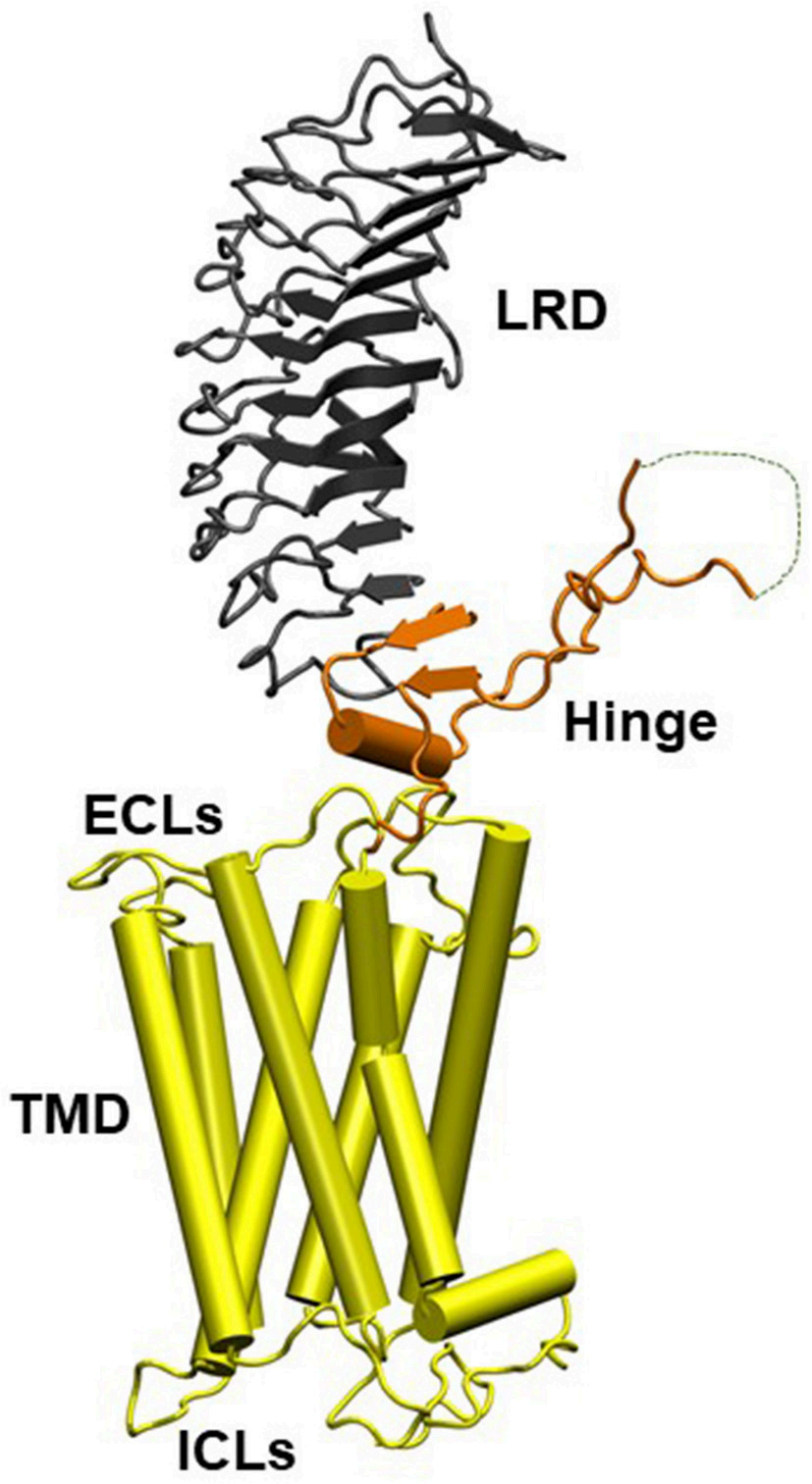
Homology model of the entire TSH holoreceptor. This model highlights the tripartite structure of the TSHR. The ectodomain, shown in gray/black, is made up of 10 leucine-rich repeat domains (LRD) characterized as a “scythe-blade” shaped structure with loops and β pleated sheets obtained from the published crystal structure (1) (PDB:3G04). The region connecting the LRD and transmembrane domain (TMD), known as the “hinge” region, has recently been crystallized for the FSH receptor (2) (PDB:4AY9) and is shown as a looped structure (orange) with a helix conformation close to the carboxyl end of the LRD. The hinge in the TSHR has an additional sequence insert and is larger than in the FSH receptor. Therefore, amino acids 305-381 are missing in the illustrated model (3) and this insert is depicted as a closed dotted loop. The TMD (yellow), with its seven helices, is depicted as cylindrical structures connected to each other by the specific TSHR intra and extracellular loops. The TMD is the region that harbors the allosteric binding pockets for the SMLs. LRD, leucine-rich domain; TMD, transmembrane domain; ECL, extracellular loops; and ICL, intracellular loops [Figure adapted from (4)].

The TSHR is a member of the class A family of G-protein coupled receptors (GPCR) with seven transmembrane helices traversing the plasma membrane and a large extracellular ectodomain. The ectodomain (ECD) is linked to a distal signal-specific domain—the hinge region—which is attached to a transmembrane domain (TMD) consisting of extracellular (ECL) and intracellular (ICL) loops (Figure 1). A partial TSHR ectodomain (residues 1–260) has been crystallized either bound to a stimulating TSHR antibody and/or a blocking TSHR antibody (1, 5) and recently in an unbound native state with stabilizing mutations. Like other GPCRs, the TSH receptor can also not exist in an ensemble of conformational states which can lead to its varied signaling potential. The review by Kleinau et al. in this collection takes a comprehensive look at the structure-function relationship of the TSHR via modeling and mutational approaches. It is now well-known that the full-length TSHR undergoes complex post translational processing (6, 7) inclusive of common protein modifications such as glycosylation and phosphorylation and even whole receptor modifications such as cleavage and multimerization (7, 8) thus resulting in a surprising variety of receptor configurations, many of which are expressed on the cell surface (9) and in some cases even shed from the cell surface (10). Although the shed receptor forms have not been conclusively demonstrated in the serum of patients with Graves' disease (GD), probably secondary to degradation, the evidence that these and other receptor structures are critical to the immunopathogenesis of GD has been well-covered in the review by Inaba et al.

**Figure 2:**
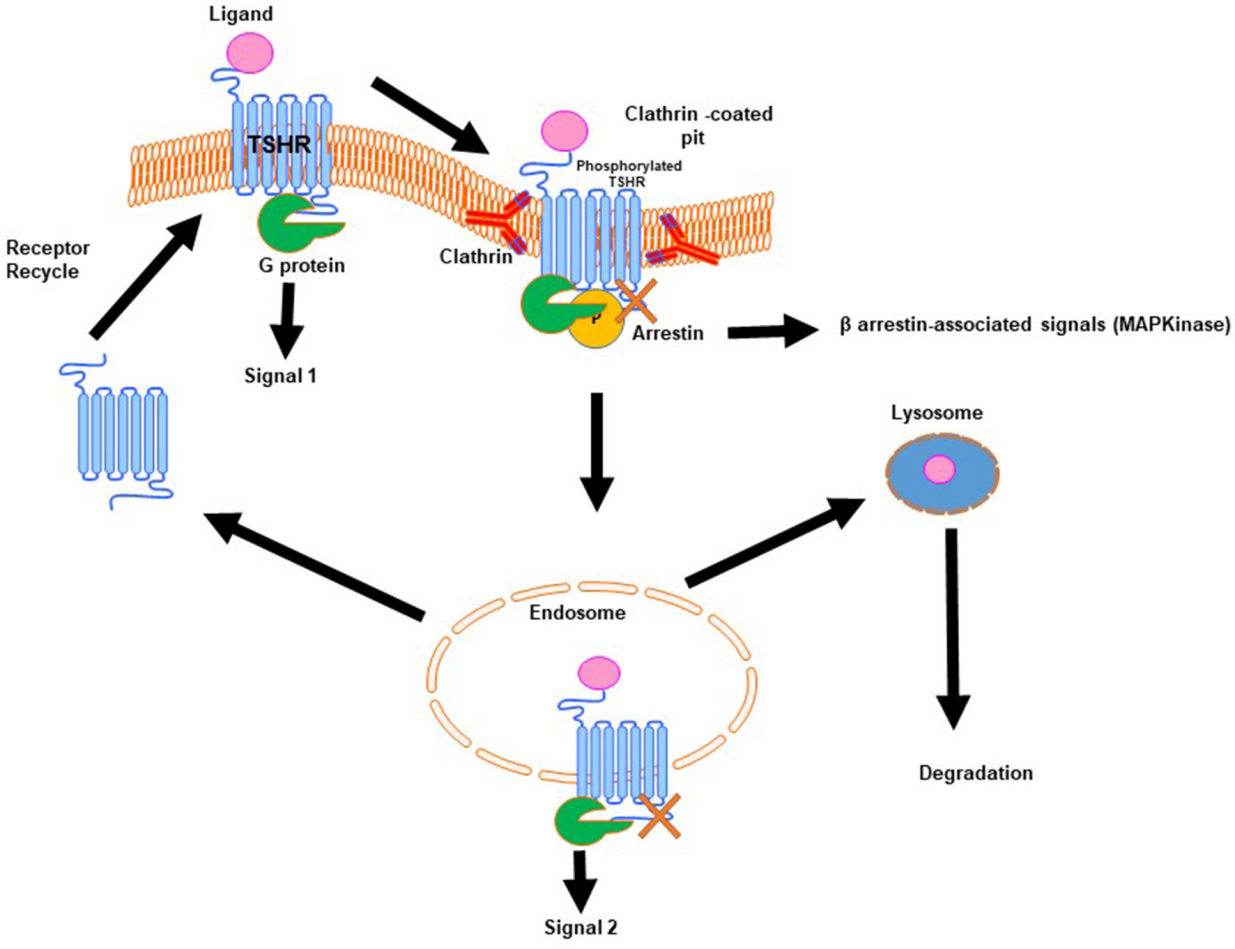
Generic life-cycle of the TSH receptor. The TSHR residing on the plasma membrane of thyrocytes on binding with its cognate ligand, TSH, is activated and in turn undergoes conformational changes to recruit and activate a G protein complex leading to a predominate wave of Gs generated cAMP (**Signal 1**). The activated receptor, after signal 1, is phosphorylated and then moved to clathrin-coated pits where β-arrestin is bound to the activated receptor. At this stage it is believed that the receptor can signal via arrestin leading to β-arrestin-associated signals. Furthermore, this activated receptor in the invaginated pits is pinched-off to form the early and late endosomes. It is described that within the endosome the receptor with its associated ligand and second messengers is capable of giving out a second wave cAMP signal (**Signal 2**). Following this the receptor can be either degraded or it enters recycling vesicles and is recycled to the plasma membrane whereas the ligand is transported to the lysosome and is degraded. This is the life that the TSHR lives on the surface of thyrocytes or any other cell where it is expressed.

Signal transduction at the TSHR is complex because of the promiscuous nature of the TSHR in engaging with different G proteins (11). In addition, the TSHR signals can be both G protein dependent and G protein independent. The TSHR has been shown to engage predominantly β-arrestin-2 for internalization (12) and arrestin-1, in human osteoblast cells, for differentiation, and MAP kinase signaling (13). In addition, it has long been known that the TSHR is involved with the IGF1/insulin receptor in thyroid cells and the “marriage” of these two receptors in fibroblasts has suggested their involvement in Graves' eye disease pathophysiology as well-reviewed by Smith et al.. The complex life cycle of GPCRs such as the TSHR (Figure 2) has also begun to be revealed showing that these types of GPCRs, after being sequestered via clathrin-coated pits or caveolin scaffolding proteins, are still able to signal after internalization. New evidence points out that these internalized receptors can lead to a “second wave” of signals from the TSHR (14). The result is that not only does the receptor come in multiple configurations but there are also multiple signal pathways that may or may not be initiated as the receptor conformation changes on ligand binding and this may continue after the receptors are internalized. The days of thinking simply of the TSH induced cyclic AMP response coming only from the surface receptors have long gone. Single-particle electron microscopy has confirmed the presence of intracellular megaplexes which consist of a GPCR bound to β-arrestin at its C terminus and a G protein complex at its core (15). The crystallization of a GPCR bound to G proteins has enhanced our understanding that ligands can stabilize different receptor conformations and that these ligand bound receptor complexes can stabilize different effector conformations leading to diversified signaling. However, such full-length receptor and G protein crystallized conformation(s) have not yet been achieved for the TSHR.

## TSHR Stimulators

The TSHR can be activated by TSH itself, or by autoantibodies which can bind to the orthosteric site(s) on the large ectodomain. In fact, activation of the TSHR has been in clinical use for many years. Semi-purified bovine TSH was originally used for short-term thyroid testing of TSHR function but proved to have too many immune related side effects in clinical practice. The clinical use of TSH was not widely adopted until the introduction of recombinant human TSH in the 1990's. This is now used for detecting thyroglobulin release from metastatic thyroid cancer and for enhancing RAI uptake into thyroid glands (16–18). The discovery of stimulating TSHR antibodies by Adams and Purves (19) demonstrated the cause of Graves' disease and helped open up the entire field of autoimmune disease. Since the discovery of TSHR autoantibodies there has been the development of clinical assays to effectively detect these antibodies in Graves' patients with improving accuracy and sensitivity. The reviews by Giuliani et al. tracing the development of TSH bioassays and by Kahaly et al. on functionality and nomenclature are interesting and important in this regard. Although the current assays for detecting these antibodies are relatively robust the solid phase assays cannot detect bioactivity and the cell based bioassays are also not ideal where high concentrations of blocking antibodies may decrease the TSHR response to stimulating antibodies. Such problems arise due to the plethora of antibodies with variable bioactivities seen in GD indicative of a wide spectrum of variable activities as discussed further below.

In recent years it been shown by several investigators that selected small molecule ligands (SML) can easily permeate the plasma membrane and allosterically activate or inhibit TSHR signals. High throughput functional screening methods led to their identification and has opened up new therapeutic potentials (20–22). Furthermore, the concept that various effectors can stabilize the TSHR in a particular conformation has opened the possibility of biased TSHR signaling as achieved with other GPCR's (23, 24).

## TSHR Antagonists

A major clinical need is for potent TSHR antagonists that can block the TSHR antibodies of hyperthyroid Graves' disease allowing us to dispense with the side effects of the common antithyroid drugs (methimazole and PTU) which deter many physicians from their long term use. A blocking human monoclonal TSHR antibody has been proposed as one method of achieving this aim (25) and results of a Phase II clinical trial are awaited. Although therapeutic antibodies have the theoretical advantage of specificity so do potential small molecule TSHR antagonists. Several groups, including our own laboratory as described by Latif et al. included in this collection (26, 27), have shown that allosteric inhibition of TSHR G protein signaling can silence the TSHR receptor. However, low potency and inadequate specificity of these SML antagonists indicate that more hurdles have to be crossed for the advancement of this approach. Peptide mimetics and aptamers to the TSHR that can either disrupt signaling via preventing G protein binding or by interfering with TSHR antigen processing are also under development and in early stage clinical trials and further data are awaited.

## Extra-Thyroidal TSHRs

At long last it is becoming widely known that the TSHR is expressed in more places than the thyroid gland and can even be found to be expressed in embryonic stem cells suggesting a role in development (11). The TSHR is expressed in fibroblasts, adipocytes, bone cells, and a variety of additional cell types (28, 29) and have, in particular, attracted a lot of attention in the retro-orbit (30–32) and bone (33, 34). This ubiquitous presence of the receptor clearly suggests that it has more functions than controlling thyroid hormone production. The role of TSHR activation and its signaling influence on adipocytes has been studied (35) and activation of the TSHR can modulate adipogenesis and fat cell phenotype further reinforced in the article by Draman et al.. The role of the TSHR in differentiation of preadipocytes into mature adipocytes from embryonic stem cells has also been shown (36) although the signals that influence this differentiation pathway are still unclear. The “Graves' Disease Triad” consists of hyperthyroidism with a dermopathy, referred to as pre-tibial myxedema, and an orbitopathy often referred to as Graves' Eye Disease and involves fibroblasts and adipocytes at both extra-thyroidal sites. Retro-orbital expression of the TSHR, in combination with IGF-1 receptors (37), expressed on the fibroblasts and adipocytes behind the eye appear to be involved in the pathogenesis of Graves' orbitopathy GO- (see Smith et al.) and serum TSHR-Ab levels tend to correlate with eye disease (38–40). IGF-1 is well-known to enhance TSH action on thyroid cells and recent studies show that blockade of the IGF-1R appears to be a useful mode of therapy for GO (41, 42) presumably by reducing stimulating TSHR-Ab-induced adipocyte proliferation and cytokine release from retro-orbital fibroblasts. Such cytokines contribute to glycosaminoglycan generation and disrupt the osmotic pressure behind the eyes causing muscle fiber damage and swelling (42, 43). Similarly, our work on TSHR expression in osteoblasts and osteoclasts has identified TSH as a potential osteoprotective molecule (33). The identification of a TSH-β subunit splice variant secreted by bone marrow macrophages may be the effector of this protective effect as discussed in detail by Baliram et al. (44).

## TSHR Antibodies

One of the unique characteristics of Graves' disease, not found in normal individuals or in the rest of the animal kingdom, is the presence of TSHR antibodies (TSHR-Ab) which are easily detectable in the vast majority of patients as discussed earlier (45). In such patients, TSHR-reactive T cells and B cells survive central and peripheral deletion and under appropriate circumstances the B cells secrete TSHR antibodies and also induce T cells to secrete pro-inflammatory cytokines (46). Hence both B cells and T cells play a central role in mediating the chronic inflammatory changes of the autoimmune diseases seen in the thyroid gland, in the retro-orbit and in the skin (19), and may be resistant to T regulatory cell (Treg) control or allowed to be active secondary to inadequate Treg function (47). Although TSHR autoantibodies represent the hallmark of GD, finding the triggers that lead to this immunological derangement has been a challenge. Genome-wide association studies have established the association of the TSHR gene specifically with GD and understanding the functional mechanism by which such polymorphisms modify the physiological processes and trigger disease by interfering with central tolerance is outlined in the review by Stefan et al.. Whatever may be the major mechanisms for these triggers we now see three varieties of TSHR-Ab that can be found in patients with autoimmune thyroid disease and in TSHR immunized rodents; stimulating, blocking, and so called “neutral” antibodies; the latter often directed at the hinge region of the TSHR ectodomain and are far from being neutral in their biological activity. Stimulating antibodies induce cyclic AMP, thyroid cell proliferation and thyroid hormone synthesis, and secretion. They bind exclusively to conformational epitopes in the TSHR ectodomain leucine rich repeat region and compete with TSH for binding. TSHR blocking antibodies compete with TSH for binding and once bound they inhibit TSH action to a variable extent. However, the degree of blocking may be profound enough that they may induce hypothyroidism although some blocking TSHR antibodies may actually behave as weak TSHR agonists. In contrast, the neutral TSHR antibodies neither block TSH binding nor block TSH action but may be involved in aberrant signal initiation and thyroid cell apoptosis (48, 49). It is important to also remember that TSHR antibodies have an important role to play in pregnancy because these antibodies cross the placenta and influence both maternal and fetal thyroid function and their biochemical and immunological aspects are well-dealt with by Bucci et al..

## Apoptosis in Graves' Disease

It is now apparent that apoptosis plays an important role in the development and perpetuation of autoimmune thyroid disease. Areas of apoptosis are recognized in thyroid tissue from patients with Hashimoto's Thyroiditis and Graves' disease (50). Subsequent studies on apoptosis have provided insight into autoimmune target destruction, indicating the involvement of death receptors and cytokine-regulated apoptotic pathways in the pathogenesis, and perpetuation of thyroid autoimmunity. There is evidence that such thyrocyte apoptosis in Graves' disease may be antibody induced (51) or T cell mediated via defects in T regulatory cells which induce an abnormal production of cytokines (52) or changes in the expression of apoptotic molecules (Fas/FasL and caspase 8) on the surface of T lymphocytes and thyroid follicular cells (53, 54). In fact, all antibody binding to the thyroid cell induces thyroid cell stress, as first shown by our own laboratory, but we have shown that some neutral antibodies induce excessive ROS accumulation leading to thyroid cell apoptosis in the absence of G-protein signaling (49, 55, 56). This antibody induced apoptosis can facilitate the breakdown of self- tolerance mechanisms in individuals with the right major histocompatibility complex (MHC) class II background in myriad ways. It could be the release of excessive cytosolic DNA fragments that can act as adjuvants/immune modulators and induce aberrant MHC II expression in thyrocytes thus inducing the release of multiple inflammatory cytokines and chemokines as seen in various animal models and well-reviewed by Luo et al. in this collection.

## The Multiplicity of TSH Receptor Forms and Responses May Explain the Graves' Disease Phenotype

With the initial discovery of the classical G-protein–coupled-receptors (GPCR) the essential mechanisms appeared at first to be straightforward. The ectodomain was responsible for hormone specificity and the intracellular domain was responsible for the cyclic AMP signal. Each receptor had a specific ligand and an expected action. The receptor for TSH was very similar to that for FSH and LH/hCG and each activated PKA and the cyclic AMP pathway. Such simplicity, however, was short lived. Firstly, the TSHR was found to have two unique inserts into the ectodomain, including one which made it subject to complex post translational processing not seen with the LHR and FSHR. Then the phenomenon of specificity cross-over reared its head. Suddenly the concept of high specificity of a hormone receptor was in doubt. For example, a number of ligands are able to bind to and activate the TSHR including hCG and LH. Stimulation of the TSHR by hCG is seen in gestational thyrotoxicosis (57) and in choriocarcinoma and a unique TSHR mutation even more highly hCG reactive has been described. With the burgeoning of our understanding into the structure of the TSHR by comparative modeling and partial crystal structures the entire field of TSHR signal transduction opened up. TSH/hCG and small molecule agonists could initiate different signals depending on the concentration of ligand available for receptor binding, the number of receptors activated, the forms of receptor (dimeric vs. monomeric) and also the orthosteric vs. the allosteric sites. Hence, we have the issue of multiple specificities and multiple signal responses indicating that an enormous number of variables are at play at just one GPCR. If we then consider Graves' disease and its multiple clinical forms which can vary from a highly localized thyroid disease to almost a systemic autoimmune diathesis much of this may be explicable by the variable forms of the receptor available for immune activation, the variable sites of TSHR expression and the multiplicity of signals that the TSHR can employ. In addition, the presence of differing proportions of high affinity TSHR-Abs with varied biological activity in patients with GD no doubt also contributes to the multiple clinical phenotypes; varying from hyperthyroidism to hypothyroidism and vice versa and with or without Graves' orbitopathy and pre-tibial myxedema.

## Conclusion

The collection of papers that form part of this special issue shows the different facets of the TSHR thus allowing us to rightly say that many roads lead from and to this GPCR. For sure the TSHR, with its structural and signaling complexity, is going to hold our scientific imagination and enthusiasm for many more years to come.

## Author Contributions

All authors listed have made a substantial, direct and intellectual contribution to the work, and approved it for publication.

### Conflict of Interest Statement

The authors declare that the research was conducted in the absence of any commercial or financial relationships that could be construed as a potential conflict of interest. The handling Editor declared a shared affiliation, though no other collaboration with the authors.
